# IMPORTANT FACTORS INFLUENCING PROSTATE CANCER SCREENING AMONG INDIGENOUS MEN

**DOI:** 10.1093/geroni/igae098.2313

**Published:** 2024-12-31

**Authors:** Kyoung Hag Lee, Jung Sim Jun

**Affiliations:** Wichita State University, Wichita, Kansas, United States; Kansas State University, Kansas State University, Kansas, United States

## Abstract

Although there exists mounting evidence of systemic barriers to prostate cancer screening (PCS) among Indigenous peoples, there is a limited understanding of the individual-level barriers to PCS. This study examined how well predisposing, need, and enabling factors predict receipt of a PCS test among Indigenous men. This cross-sectional study recruited a total of 191 Indigenous men aged 40 and above, following the recommendation of the American Cancer Society by using the purposive sampling method in 2014. The study’s dependent variable was the receipt of a PCS. Independent variables included three predisposing, two need, and five enabling factors. Participants’ mean age was 51.58 years, ranging from 40 to 95, and 33.3% were married. The hierarchical logistic regression showed that married participants were 3.82 times more likely than those who never married to receive a PCS. Participants with a personal cancer history were 11.84 times more likely than those without a personal cancer history to receive a PCS. Participants who reported having access to medical care were 9.14 times more likely than those without access to medical care to receive a PCS. Additionally, participants who had heard about the prostate cancer exam were 21.44 times more likely than those who had not heard about the prostate cancer exam to receive a PCS. Social work practitioners or health care providers need to consider developing and providing appropriate prostate cancer prevention programs, family-based intervention programs, a support system for selecting an appropriate place for medical care, and prostate cancer screening education and assistance programs.

